# Isolation and characterization of a bacteriophage phiEap-2 infecting multidrug resistant *Enterobacter aerogenes*

**DOI:** 10.1038/srep28338

**Published:** 2016-06-20

**Authors:** Erna Li, Xiao Wei, Yanyan Ma, Zhe Yin, Huan Li, Weishi Lin, Xuesong Wang, Chao Li, Zhiqiang Shen, Ruixiang Zhao, Huiying Yang, Aimin Jiang, Wenhui Yang, Jing Yuan, Xiangna Zhao

**Affiliations:** 1 College of Food Science, South China Agricultural University, Guangzhou, 510642, China; 2 Institute of Disease Control and Prevention, Academy of Military Medical Sciences, Beijing, 100071, China; 3 College of Food Science, Henan Institute of Science and Technology, Xinxiang, 453003, China; 4 State Key Laboratory of Pathogen and Biosecurity, Beijing Institute of Microbiology and Epidemiology, Beijing, 100071, China; 5 Key Laboratory of Risk Assessment and Control for Environment and Food Safety, Tianjin Institute of Health and Environmental Medicine, Tianjin, 300050, China

## Abstract

*Enterobacter aerogenes* (Enterobacteriaceae) is an important opportunistic pathogen that causes hospital-acquired pneumonia, bacteremia, and urinary tract infections. Recently, multidrug-resistant *E. aerogenes* have been a public health problem. To develop an effective antimicrobial agent, bacteriophage phiEap-2 was isolated from sewage and its genome was sequenced because of its ability to lyse the multidrug-resistant clinical *E. aerogenes* strain 3-SP. Morphological observations suggested that the phage belongs to the *Siphoviridae* family. Comparative genome analysis revealed that phage phiEap-2 is related to the *Salmonella* phage FSL SP-031 (KC139518). All of the structural gene products (except capsid protein) encoded by phiEap-2 had orthologous gene products in FSL SP-031 and *Serratia* phage Eta (KC460990). Here, we report the complete genome sequence of phiEap-2 and major findings from the genomic analysis. Knowledge of this phage might be helpful for developing therapeutic strategies against *E. aerogenes*.

*Enterobacter aerogenes* is a gram-negative bacterium of the Enterobacteriaceae family^1^. This bacterium is widely found in the human gastrointestinal tract and environment^2^. *E. aerogenes* has been reported to be an important opportunistic pathogen for humans^3^, and it is resistant to multiple antibiotics that are normally used to treat infections caused by *Enterobacter*^4^. It causes hospital-acquired infections such as pneumonia, bacteremia, urinary tract infection, surgical site infection, and meningitis^2^. Phages by their very nature would seem to be good candidates for antibacterial therapy^5^. Although the use of phages for therapeutic purposes has raised concerns over the potential for immunogenicity, restriction/modification, rapid toxin release by lytic action, development of bacteria resistance and so on^5^,^6^,^7^,^8^, phage therapy was still actively pursued. For example, a *Siphoviridae* phage 1535 against *K. pneumoniae* has been reported to have great potential for treating pneumonia and other infections caused by *K. pneumoniae*^9^, and the application of the *Yersinia* phage PY100 for the control of *Y. enterocolitica* at the post-harvest level seems to be promising^10^. To date, there are only two reported *E. aerogenes* phages, F20 (JN672684)^4^ which is a member of the *Siphoviridae* family of T1-like viruses, and an unclassified phage UZ1^11^. To expand the repertoire of phages available for targeting clinically relevant *E. aerogenes*, a novel phage (phiEap-2) against *E. aerogenes* was isolated from hospital sewage, and the biology and genomics of the phage were characterized. This phage has specific lytic activity against a carbapenem-nonsusceptible *E. aerogenes* strain, 3-SP, which contains an NDM-1 carbapenemase-producing plasmid designated as p3SP-NDM and is resistant to multiple β-lactam antibiotics including imipenem and meropenem^2^. The phage is a member of the *Siphoviridae* family; the morphology, one-step growth curve, stability studies and complete genome sequence of the phage were determined.

## Results and Discussion

### Morphology

**Figure 1:**
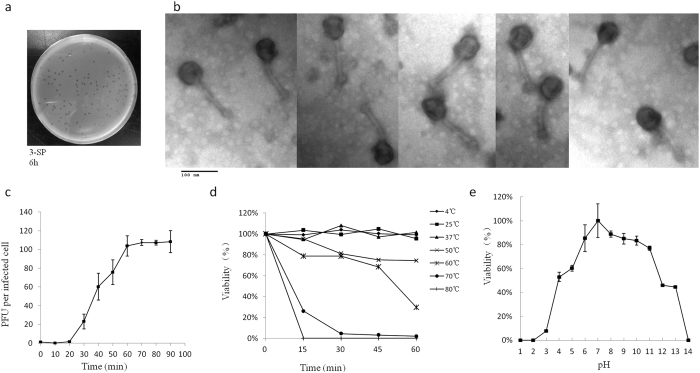
Isolated enterobacteria phage phiEap-2. (**a**) Plaques of phage phiEap-2 on *E. aerogenes* 3-SP. (**b**) Transmission electron micrograph (TEM) of phage phiEap-2 at × 150 000. The bar indicates 100 nm. (**c**) One-step growth curve of phiEap-2. Phages were grown in an exponential phase culture of *E. aerogenes*. (**d**) Stability of phage phiEap-2 at different temperatures. (**e**) Stability of phage phiEap-2 at different pH.

Phage phiEap-2 produced large, clear, round plaques of 1–2 mm in diameter on a lawn of *E. aerogenes* 3-SP (Fig. 1a). The phage was purified and examined by TEM after negative staining (Fig. 1b). The phage had a capsid that was 55 nm in diameter and a non-contractile tail that was about 117 nm long and 10 nm in diameter; these morphological features indicate that this virus belongs to the *Siphoviridae* family. Host range tests suggested that phiEap-2 was specific for *E. aerogenes* (Table 1).

### Life cycle parameters

Multiplication parameters of phage phiEap-2 were determined using one-step growth curve conditions (Fig. 1c). The latent period, defined as the time interval between the adsorption and the beginning of the first burst, was about 25 min. A burst time of 60 min and average burst size of 100 plaque-forming units (pfu)/cell, which was calculated as the ratio of the final count of liberated phage particles to the initial count of infected bacterial cells during the latent period, were observed. The thermal stability of phage phiEap-2 was determined at different temperatures from 4 to 80 °C (Fig. 1d). No significant loss in the phage titer was observed from 4 to 37 °C. Phage phiEap-2 had a titer reduction at 50 °C, and the titers dramatically decreased at 60 °C. Stability of phage phiEap-2 with different pH were also conducted (Fig. 1e). The phage was stable over a broad range of pH from 6 to 11. A significant reduction in phage titre was observed when it was extremely acidic (pH 3) or basic (pH 14). It was noticed that still nearly half of the phages existed at pH 4 or pH 13. The results suggested that the phage appears to tolerate better basic than acidic conditions.

### General features of the phiEap-2 genome

**Figure 2:**
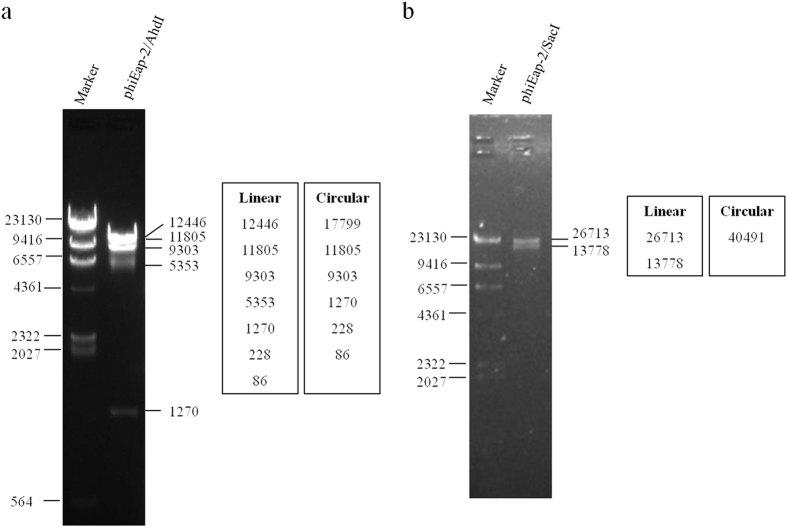
Restriction fragment length polymorphism analysis of phiEap-2 DNA. Genomic DNA from phage phiEap-2 was digested with the enzymes indicated (*AhdI* and *SacI*) and run on an agarose gel (0.7%). The length of fragments generated by digestion of the linear genome or the circular genome was showed on the right side of the electrophoresis map.

**Figure 3:**
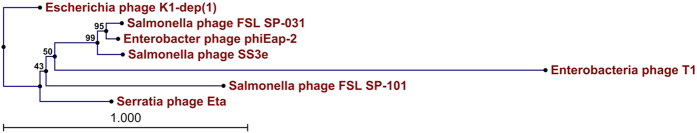
Phylogenetic tree based on large terminase subunits of selected bacteriophages. The large terminase subunits were compared using the ClustalW program, and the phylogenetic tree was generated using the neighbour-joining method and 1000 bootstrap replicates. FSL SP-031, GenBank accession no. NC_021775; phiEap-2, GenBank accession no. KT287080; Eta, GenBank accession no. KC460990; FSL SP-101, GenBank accession no. KC139511; K1-dep(1), GenBank accession no. GU196278; SS3e, GenBank accession no. NC_006940; T1, GenBank accession no. AY216660.

**Figure 4:**
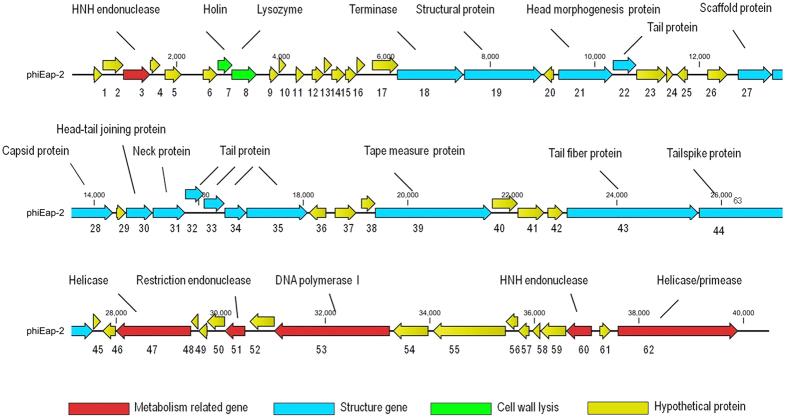
Genomic structure of phiEap-2. The genome map was performed using the CLC Main Workbench, version 6.1.1 (CLC bio, Aarhus, Denmark). Arrows represent predicted ORFs, the direction of the arrow represents the direction of transcription. Different colors denote different functional groups of bacteriophage genes.

The genome of phage phiEap-2 is 40,491 bp in length with a 51.95% GC content. When the original sequencing was completed, the assembly of the random library of sequences yielded a closed, circular genome. However, based on the genome sequence analysis and the length of the fragments generated by digestion with *AhdI* (Fig. 2a) and *SacI* (Fig. 2b), the genome is linear rather than circular. Sixty-two open reading frames (ORFs) were predicted in the phage genome. Twenty-three ORFs were functionally annotated, and thirty-nine ORFs were annotated as hypothetical proteins. The orientation of genome annotation was chosen so that most genes (71%) were on the plus strand. The capsid and tail genes were identified, as well as representatives of the DNA replication, packaging, and lysis proteins. Comparative genome analysis of phiEap-2 with existing phages supports the hypothesis that phiEap-2 has no similarity to the previously published *E. aerogenes* phage F20^4^, but it is related to the previously sequenced *Salmonella* phage FSL SP-031 (KC139518)^12^ (Fig. 3). Both of phiEap-2 and FSL SP-031 belong to the *Siphoviridae* family of viruses. This family is characterized by having a double-stranded DNA genome, an isometric head, and a long, non-contractile tail. FSL SP-031 encoded 59 predicted proteins despite a genome of 42, 215 bp, which was larger than that of phiEap-2. For comparison, the GC content of FSL SP-031 is 51.3%. PhiEap-2 and FSL SP-031 shared thirty-five orthologous genes; the amino acid identities for these genes are shown in Table 2. The phiEap-2 phage has a similar gene arrangement that was observed in other *Siphoviridae* phages^13^: genes for head and tail assembly were arranged together with the head genes 5′ to the tail genes. phiEap-2 genes were categorized into three functional groups according to the homology search-based annotation of functional genes (Fig. 4). ORF information, such as the position of genes, protein length, directions of transcription, size, function, and homology between phiEap-2 genes and other phage-related genes, is shown in Table 2.

### DNA metabolism

At least six genes in the phiEap-2 genome that play a role in nucleotide metabolism were identified, including a helicase, a replicative helicase-primase, a restriction endonuclease, a DNA polymerase, and two alleles of HNH endonuclease. *gp47* encodes a helicase consisting of an SNF2 family N-terminal domain (pfam00176) and a helicase-conserved C-terminal domain (pfam00271). A BLASTP search of the helicase revealed significant identity with the FSL SP-031 orf2 (96%). *gp62*, which encodes a replicative helicase-primase containing an AAA_25 (pfam13481) domain and a hexameric replicative helicase RepA region, showed 75% identity with FSL SP-031 orf16. The restrictive endonuclease encoded by *gp51* of phiEap-2 showed homology to FSL SP-031 orf3 (65%) and contained a VRR-NUC domain (pfam08774). *gp53*, which encodes a DNA polymerase I containing a 3′-5′ exonuclease domain (pfam01612) and a DNA polymerase family A domain (pfam00476), showed similarity to FSL SP-031 orf5 (74%). Proteins containing an HNH motif bind to nucleic acids and possess endonuclease activity^14^. The two HNH endonucleases encoded by *gp60* and *gp3* were homologous except for some amino acid changes; both of the proteins consist of an AP2 domain (pfam00847) and an HNH endonuclease domain (pfam13392), and they shared 67% and 49% identity with *Salmonella* phage SETP7 (NC_022754) *gp42*, respectively. The HNH endonucleases are unique to phiEap-2 compared with FSL SP-031.

### Cell wall lysis-related genes

PhiEap-2 has a holin-encoding gene (*gp7*) immediately upstream of the lysozyme gene (*gp8*), suggesting that it may use a holin-dependent lytic mechanism. The lysis genes are located to the left of the terminase gene. As in many other double-stranded DNA phages, *gp7*, which encodes a predicted holin composed of 94 amino acids, and *gp8*, which encodes a lysozyme with 80% homology to the FSL SP-031 orf24, seemed to be involved in the holin-endolysin system.

### Structural proteins

The arrangement of genes encoding phiEap-2 structure assembly proteins followed the conserved synteny and gene orders of *Siphovirus*^13^. Entire structural gene products encoded by phiEap-2, except for capsid protein, had orthologous gene products in FSL SP-031 and Eta (Table 2). The observation revealed a significant evolutionary relationship between FSL SP-031 and Eta. BLASTP analysis of *gp18* of phiEap-2 showed significant homology to the C-terminal sequence of the Eta terminase large subunit. Different from Eta, whose terminase small subunit was located immediately upstream of the terminase large subunit, the phiEap-2 phage has only one terminase subunit. It is necessary to point out that *gp28*, especially the region that encodes the major capsid protein of phiEap-2, revealed extensive conservation with *Escherichia* phage K1-dep(1) and had no sequence similarity with FSL SP-031 or Eta, although coat proteins of FSL SP-031 and Eta were located in the same position of the genome. Five ORFs (*gp22* and *gp32*–*35*) were annotated as tail proteins, and they had amino acid identities with FSL SP-031 orthologous genes that ranged from 58% to 90%. Tail fibers and tailspikes are appendages in the phage tail that facilitate the initial binding of the phage to the bacterial host and have a role in host specificity^12^. The tail fiber protein encoded by *gp43* shares 60% amino acid identity with FSL SP-031 orf58. The tailspike protein encoded by *gp44* has a mosaic nature: the N terminus displayed similarity (59%) to FSL SP-031 orf59, and the C-terminus more closely resembles a hypothetical protein of *E. aerogenes*. This observation is consistent with previous reports that these genes often show diversity due to recombination^12^ and also may suggest that these genes are linked to the evolution of host specificity^12^. phiEap-2 *gp39*, which encodes a tape measure protein (TMP) and is the largest gene in the genome, contains a TMP_2 domain (pfam06791) in the N-terminal end. TMP is important for the assembly of phage tails, is involved in tail-length determination, and corresponds to the length of the phage tail^15^,^16^. Comparisons of the phiEap-2 and FSL SP-031 proteins revealed 65% identity, and the N and C termini were highly similar.

## Concluding remarks

The emergence of multiple antibiotic resistant *E. aerogenes* strains has limited the use of antibiotics to control this pathogen. The phiEap-2 genome does not encode any phage lysogeny factors, toxins, antibiotic resistance genes or pathogen-related genes, indicating that phiEap-2 may be considered a virulent phage with no side effects. Stability is the primary requirement when considering phage for commercial use^4^. Here, we isolated a phage from sewage, reported its sequence analysis, and presented data relating to its initial characterization. TEM showed that the phage belonged to the *Siphoviridae* family; these phage possess isometric heads and long, non-contractile tails. The genome of the phage was found to be double-stranded DNA and showed sequence homology to *Salmonella* phage FSL SP-031. The structural gene module showed a degree of sequence conservation with FSL SP-031 and *Serratia* phage Eta. The evolution of phage is thought to involve the exchange of functional modules via the loss or acquisition of genetic material by recombination between phage and also between phage and their hosts. The evolutionary advantage of this genetic recombination is thought to assist phage in their permanent adaptation to changing environmental conditions or in their quest to infect new hosts^17^. Although there is significant diversity among phages^18^, the structural gene module was found at an equivalent location in the genomes of the three members of the Siphoviridae (phiEap-2, FSL SP-031 and Eta), indicating an evolutionary connection between these phage. Characterization of phage phiEap-2 will assist in its exploitation as a therapeutic candidate against *E. aerogenes* and as a biocontrol agent to prevent contamination by *E. aerogenes*. However, considering the development of bacterial resistance to phage and the fact that relatively few candidate phage that lyse *E. aerogenes* have been properly characterized, there is demand for the isolation of novel *E. aerogenes* phage to expand the repertoire of phage available for targeting clinically significant *E. aerogenes*. Furthermore, an increased repertoire of available phage may allow for the development of multi-phage cocktails that may be broadly effective against a wide range of bacterial targets. In our future work, this possibility will be explored. In this study, the phenotypic features and genetic properties of the phage were examined, providing the basis for future therapeutic work. The sequenced phage might also be used in investigations of phage–bacterium interactions.

## Methods

### Phage isolation

*E. aerogenes* strains were grown in Luria–Bertani (LB) broth medium at 37 °C. Multidrug-resistant *E. aerogenes* strain 3-SP was used as a host strain for phage isolation from Bejing hospital sewage. The sewage samples were centrifuged at 12,000 × *g* for 10 min to remove the solid impurities. The supernatants were filtered through a 0.22-μm pore-size membrane filter to remove bacterial debris. Filtrate (300 μL) was added to 5 ml LB broth medium and mixed with 200 μL *E. aerogenes* culture (optical density at 600 nm, OD_600_ = 0.6) to enrich the phage at 37 °C for 8 h. Then, the culture was centrifuged at 12,000 × *g* for 10 min, and the supernatant was filtered with a 0.22-μm pore-size membrane filter to remove the residual bacterial cells. Filtrate diluted into medium (100 μL) was mixed with 300 μL *E. aerogenes* in LB culture (OD_600_ = 0.6) and 3 ml molten top soft nutrient agar (0.7% agar), which was then overlaid on solidified base nutrient agar (1.5% agar)^19^. Following incubation for 8 h at 37 °C, clear phage plaques were picked from the plate. The phage titer was determined using the double-layered method.

### Purification of the phage

To prepare phiEap-2 for transmission electron microscopy (TEM) studies, cell debris from 400 ml *E. aerogenes* strain 3-SP infected with phiEap-2 was pelleted by low-speed centrifugation (8,500 × *g*, 20 min, 4 °C). Phage particles were precipitated with 1 M NaCl and 10% polyethylene glycol (PEG) 8000 at 4 °C with stirring for 60 min. The precipitated phage particles were harvested by low-speed centrifugation (8,500 × *g*, 20 min, 4 °C). Phage particles were resuspended in TM buffer (50 mM Tris-HCl [pH 7.8], 10 mM MgSO_4_) and extracted with an equal volume of chloroform. After low-speed centrifugation (3,000 × *g*, 15 min, 4 °C), the aqueous phase was sedimented at about 25,000 × *g* for 60 min^20^.

### Electron microscopy

Phage particles were negatively stained with 2% (wt/vol) phosphotungstic acid, pH 7. Stained particles were observed in a Philips EM 300 electron microscope operated at 80 kV. Dimensions were measured on photographic prints at a final magnification of 150,000×^21^.

### One-step growth curve

A mid-exponential-phase culture (10 ml) of *E. aerogenes* strain 3-SP (OD_600_ = 0.4 to 0.6) was harvested by centrifugation and resuspended in 0.25 volume of fresh LB (ca. 10^9^ colony-forming units/ml). 10^6^–10^7^ pfu/ml phage was added at a multiplicity of infection of 0.1 and allowed to adsorb for 5 min at room temperature. The mixture was then centrifuged at 10,000 rpm for 10 min at room temperature, pelleted cells were resuspended in 10 ml LB, and incubation was continued at 37 °C. Samples were taken at 10-min intervals for 80 min. The samples were immediately diluted and plated for phage titration^22^. Burst size is calculated as the ratio of the final count of liberated phage particles to the initial count of infected bacterial cells during the latent period. Measurement of phage’s latent-period duration was accomplished by detecting the delay between phage adsorption of a bacterium and the liberation of phage virions^23^,^24^,^25^.

### Stability

A temperature-controlled incubator or water bath was used to determine the stabilities at different temperatures or pH. Briefly, a 1.5-ml tube containing equal volumes of phage (2.5 × 10^8 ^pfu/ml) were incubated at a specified temperature or pH. After treatment, the tube was cooled slowly and placed in an ice water bath; samples were assayed to determine surviving phages. The results were expressed as a percentage of the initial viral counts. Each assay was performed as three repetitions and the values represented are the means.

### Host range determination

The lytic activity of phiEap-2 was tested against 14 species as determined by standard spot tests^26^. The strains to be tested were grown overnight in LB. Briefly, 10 μL purified phage suspension containing approximately 10^8^ pfu/ml were spotted in the middle of a lawn of bacteria and left to dry before overnight incubation. Bacterial sensitivity to a bacteriophage was established by bacterial lysis at the spot where the phage was deposited. Each strain was tested three times at 37 °C. Efficiency of plating (EOP), the ratio of pfu/ml obtained with an assay host to the pfu/ml obtained with the isolation host, was calculated using the double layer plaque method. Assay host refers to the tested *E. aerogenes* clinical isolates, and isolation host refers to the *E. aerogenes* isolate 3-SP with which we initially isolated the phage.

### Preparation of phage DNA

The precipitated phage were resuspended in SM buffer^27^ and purified by Caesium chloride (CsCl) gradient ultracentrifugation based on a method previously described^28^. The gradients were prepared in Beckman SW32.1 tubes by subsequently underlaying 1.5 ml of each 1.33, 1.45, 1.6 and 1.7 g/cm^3^ CsCl solution. Phages were gently added on top of the 1.33 g/cm^3^ CsCl. The tubes were centrifuged at 140,000 g for 3 h at 4 °C. The opalescent phage band was collected using a glass pasteur pipette and dialysed (1000 kDa MWCO), twice for 2 h and once overnight, against 250 volumes (500 ml) of SM buffer to remove CsCl. Phages were concentrated and the titre was determined. Purified phage titres were 1 × 10^11^ pfu/ml and stored in the dark at 4 °C 28. DNA of high titer suspensions (10^10^ pfu/ml) of filtered phage lysate was extracted with the phenol-chloroform (24:1, vol/vol) method and precipitated with ethanol. The samples were analyzed on 0.7 to 1.0% agarose gels.

### Genome sequencing and computational analysis

The purified phage phiEap-2 genomic DNA was sequenced using an Illumina HiSeq 2500 sequencer. The preparation of the library was done using a KAPA Hyper Prep Kit Illumina platforms following the manufacturer’s instructions. The assembly used 7,907,550 reads, or 988.4 MB, of raw data to give a 24410 × coverage of the genome. The reads were assembled using SSAKE (v3.8) assembly software. The final assembled sequence was searched against the current protein and nucleotide databases (http://www.ncbi.nlm.nih.gov/) using the basic local alignment search tool (BLAST)^29^. Protein BLAST (BLASTP) (http://www.ncbi.nlm.nih.gov/BLAST/) was used to identify putative homologies and proteins sharing similarities with predicted phage proteins. The CLC Main Workbench, version 6.1.1 (CLC bio, Aarhus, Denmark), was used for genome annotation. Simulation of the restriction enzyme mapping of the phiEap-2 genome sequence was carried out using the software package DNAStar. The phiEap-2 DNA was digested by selected restriction endonucleases (*AhdI* and *SacI*, purchased from New England Biolabs, Ipswich, MA, USA). For a reaction system of 20 μL, 10 units of the restriction endonuclease and 200 ng of phiEap-2 DNA were used. The mixture was incubated at 37 °C for 120 min and then used to perform agarose gel electrophoresis. Agarose gel electrophoresis was subsequently performed to separate the restriction fragments. Phylogenetic analysis with the published genome sequences of related phages was performed using ClustalW. Multiple sequence alignment was carried out using Mauve software.

## Additional Information

**Accession code**: The annotated genome sequence for the phage phiEap-2 was deposited in the NCBI nucleotide database under the accession number KT287080.

**How to cite this article**: Li, E. *et al.* Isolation and characterization of a bacteriophage phiEap-2 infecting multidrug resistant *Enterobacter aerogenes.* *Sci. Rep.* **6**, 28338; doi: 10.1038/srep28338 (2016).

## Figures and Tables

**Table 1:** Host range infection of the phage phiEap-2 and efficiency of plating.

Species	ID	Infection	Efficiency of plating (EOP)
*E. aerogenes*	3-SP	+	1.00
*E. aerogenes*	201316724	+	1.12
*E. aerogenes*	2015-301	+	0.84
*E. aerogenes*	13208	−	0
*E. aerogenes*	A29864	−	0
*E. aerogenes*	A36179	−	0
*E. aerogenes*	AH10	−	0
*E. aerogenes*	AH12	+	0.97
*E. aerogenes*	AH13	+	0.85
*E. aerogenes*	AH14	+	0.60
*E. aerogenes*	AH15	+	0.20
*E. aerogenes*	AH17	+	0.51
*E. aerogenes*	AH18	+	1.03
*E. aerogenes*	AH2	−	0
*E. aerogenes*	AH20	+	0.18
*E. aerogenes*	AH21	+	1.01
*E. aerogenes*	AH22	+	0.59
*E. aerogenes*	AH24	−	0
*E. aerogenes*	AH25	+	0.03
*E. aerogenes*	AH28	+	1.12
*E. aerogenes*	AH29	+	0.63
*E. aerogenes*	AH3	+	0.23
*E. aerogenes*	AH30	+	0.29
*E. aerogenes*	AH32	+	0.80
*E. aerogenes*	AH33	−	0
*E. aerogenes*	AH34	+	0.61
*E. aerogenes*	AH36	+	0.75
*E. cloacae*	T5282	−	0
*E. cloacae*	TI3	−	0
*E. sakazakii*	45401	−	0
*E. sakazakii*	45402	−	0
*Serratia marcescens*	wk2050	−	0
*S. marcescens*	201315732	−	0
*S. marcescens*	wj-1	−	0
*S. marcescens*	wj-2	−	0
*S. marcescens*	wj-3	−	0
*Escherichia coli*	ATCC 25922	−	0
*Klebsiella pneumoniae*	ATCC BAA-1706	−	0
*Achromobacter xylosoxidans*	A22732	−	0
*Leclercia adcarboxglata*	P10164	−	0
*Raoultella ornithinolytica*	YNKP001	−	0
*Stenotrophomonas maltophilia*	9665	−	0
*Citrobacter freundii*	P10159	−	0
*Vibrio parahaemolyticus*	J5421	−	0
*Pseudomonas aeruginosa*	PA01	−	0
*Acinetobacter baumannii*	N1	−	0
*Shigella sonnei*	#1083	−	0

^−^absent; ^+^present.

**Table 2:** Phage phiEap-2 gene annotations.

ORFs	Strand	Nucleotide position	Length (aa)^a^	Conserved Protein Domain Family	Best match (%)	Vs FSL SP-031	Vs Eta	Function
ORF1	+	409–582	58					
ORF2	+	582–983	133		FSL SP-031 orf18 (54%)	orf18 (54%)		
ORF3	+	976–1494	172	pfam13392; pfam00847; PHA00280	SETP7 orf42 (49%)			HNH endonuclease
ORF4	+	1491–1688	65		FSL SP-031 orf19 (89%)	orf19 (89%)		
ORF5	+	1770–2096	108	pfam11753	FSL SP-031 orf20 (82%)	orf20 (82%)		
ORF6	+	2500–2781	93		SETP7 orf57 (53%)	orf22 (53%)		
ORF7	+	2783–3067	94		SETP7 orf58 (57%)	orf23 (45%)		Class I holin
ORF8	+	3048–3533	161	cd00737; COG3772; pfam00959	FSL SP-031 orf24 (80%)	orf24 (80%)		Lysozyme
ORF9	+	3772–3957	62					
ORF10	+	3954–4097	47		FSL SP-031 orf25 (93%)	orf25 (93%)		
ORF11	+	4275–4445	56	pfam10930	FSL SP-031 orf26 (87%)	orf26 (87%)		
ORF12	+	4585–4821	78		FSL SP-031 orf27 (49%)	orf27 (49%)		
ORF13	+	4818–4973	52		FSL SP-031 orf28 (50%)	orf28 (50%)		
ORF14	+	4960–5220	86		pYD38-A orf74 (74%)			
ORF15	+	5223–5444	73	pfam06322	FSL SP-031 orf29 (72%)	orf29 (72%)		
ORF16	+	5441–5608	56					
ORF17	+	5738–6241	167		FSL SP-031 orf31 (60%)	orf31 (60%)		
ORF18	+	6216–7487	423	TIGR01547; pfam03237	FSL SP-031 orf32 (89%)	orf32 (89%)	orf40 (57%)	Terminase large subunit
ORF19	+	7500–8987	495	pfam13264	K1-dep(1) orf2 (80%)	orf33 (77%)	orf42 (65%)	Structural protein
ORF20	−	9014–9217	68					
ORF21	+	9302–10345	347	TIGR01641; pfam04233; COG2369	FSL SP-031 orf34 (91%)	orf34 (91%)	orf43 (66%)	Head morphogenesis protein
ORF22	+	10345–10794	149	pfam07679	FSL SP-031 orf35 (67%)	orf35 (67%)	orf44 (55%)	Tail protein
ORF23	+	10794–11363	189		FSL SP-031 orf35 (35%)	orf35 (35%)	orf44 (35%)	
ORF24	+	11367–11501	44		BA3 orf20 (66%)			
ORF25	−	11567–11779	71		Acj61 p098 (39%)			
ORF26	+	12153–12542	129		FSL SP-031 orf37 (61%)	orf37 (61%)		
ORF27	+	12736–13386	216		Eta orf50 (64%)	orf40 (54%)	orf50 (64%)	Scaffold protein
ORF28	+	13393–14361	322		K1-dep(1) orf8 (93%)			Major capsid protein
ORF29	+	14422–14607	61		K1-dep(1) orf 9 (56%)			
ORF30	+	14611–15117	168	PRK00007	FSL SP-031 orf45 (75%)	orf45 (75%)	orf54 (70%)	Head-tail joining protein
ORF31	+	15120–15743	207		FSL SP-031 orf46 (73%)	orf46 (73%)	orf55 (72%)	Neck protein
ORF32	+	15740–16099	119		FSL SP-031 orf47 (82%)	orf47 (82%)	orf56 (77%)	Tail protein
ORF33	+	16096–16494	132	pfam04883	FSL SP-031 orf48 (58%)	orf48 (58%)	orf57 (51%)	Tail protein
ORF34	+	16494–16910	138	pfam13554	FSL SP-031 orf49 (90%)	orf49 (90%)	orf58 (81%)	Tail protein
ORF35	+	16913–18085	390		FSL SP-031 orf50 (86%)	orf50 (86%)	orf59 (77%)	Tail protein
ORF36	−	18108–18440	111		FSL SP-031 orf51 (41%)	orf51 (41%)		
ORF37	+	18602–19018	138		FSL SP-031 orf52 (82%)	orf52 (82%)	orf61 (57%)	
ORF38	+	19108–19380	90		FSL SP-031 orf53 (81%)	orf53 (81%)	orf62 (53%)	
ORF39	+	19373–21607	744	pfam06791; PTZ00121	FSL SP-031 orf54 (65%)	orf54 (65%)	orf63 (53%)	Tail tape measure protein
ORF40	+	21610–22104	164		Eta orf64 (75%)	orf55 (64%)	orf64 (75%)	
ORF41	+	22101–22613	170	pfam08875	Eta orf66 (78%)	orf56 (69%)	orf66 (78%)	
ORF42	+	22673–22975	100		Eta orf67 (64%)	orf57 (58%)	orf67 (64%)	
ORF43	+	23041–25557	838	COG4733	FSL SP-031 orf58 (60%)	orf58 (60%)	orf68 (60%)	Tail fiber protein
ORF44	+	25570–27558	662	pfam12708; pfam13229; TIGR04247	Eta orf69 (37%)	orf59 (24%)	orf69 (37%)	Tailspike protein
ORF45	+	27555–27707	50					
ORF46	−	27736–27993	86	pfam13600; cd14812	FSL SP-101 orf1 (72%)	orf1 (68%)		
ORF47	−	27996–29432	478	cd00046; pfam00271; pfam00176; smart00487; COG0553	FSL SP-031 orf2 (96%)	orf2 (96%)		Helicase
ORF48	−	29429–29572	48					
ORF49	−	29572–29745	58					
ORF50	−	29736–30080	114	PHA00527	Marshall orf108 (47%)			
ORF51	−	30077–30469	130	pfam08774	FSL SP-031 orf3 (65%)	orf3 (65%)		Restriction endonuclease
ORF52	−	30552–31031	159	pfam13392; pfam00847; PHA00280				
ORF53	−	31021–33234	737	cd08642; smart00482; pfam00476; pfam01612; PRK14975; TIGR00593; COG0749	FSL SP-031 orf5 (74%)	orf5 (74%)		DNA polymerase I
ORF54	−	33293–33973	226	pfam10991	FSL SP-031 orf7 (85%)	orf7 (85%)		
ORF55	−	34058–35452	464	pfam10926	FSL SP-031 orf9 (72%)	orf9 (72%)		
ORF56	−	35452–35688	78		FSL SP-031 orf11 (58%)	orf11 (58%)		
ORF57	−	35685–35906	74					
ORF58	−	35955–36113	53					
ORF59	−	36117–36608	163	PRK13108; TIGR00457	FSL SP-031 orf14 (42%)	orf14 (42%)		
ORF60	−	36611–37099	162	pfam13392; pfam00847; PHA00280	SETP7 orf42 (67%)			HNH endonuclease
ORF61	+	37244–37465	73	cd00093; pfam13443; smart00530; COG3655; PRK09706; COG1396	FSL SP-031 orf15 (39%)	orf15 (39%)		
ORF62	+	37594–39897	767	pfam13481; cd01125	FSL SP-031 orf16 (75%)	orf16 (75%)		Replicative helicase/primease

^a^amino acids.
