# Corrigendum: Isolation and characterization of a bacteriophage phiEap-2 infecting multidrug resistant *Enterobacter aerogenes*

**DOI:** 10.1038/srep46805

**Published:** 2017-05-09

**Authors:** Erna Li, Xiao Wei, Yanyan Ma, Zhe Yin, Huan Li, Weishi Lin, Xuesong Wang, Chao Li, Zhiqiang Shen, Ruixiang Zhao, Huiying Yang, Aimin Jiang, Wenhui Yang, Jing Yuan, Xiangna Zhao

Scientific Reports 6: Article number: 2833810.1038/srep28338; published online: 06 20 2016; updated: 05 09 2017

In the original version of this Article, Wenhui Yang was incorrectly listed as being affiliated with ‘Key Laboratory of Risk Assessment and Control for Environment and Food Safety, Tianjin Institute of Health and Environmental Medicine, Tianjin, 300050, China’. The correct affiliation is listed below:

State Key Laboratory of Pathogen and Biosecurity, Beijing Institute of Microbiology and Epidemiology, Beijing, 100071, China.

In addition, Table 1 previously contained the results of the host range of an incorrect *Enterobacter aerogenes* phage. The correct Table 1 appears below.

These errors have now been corrected in the HTML and PDF versions of the Article.

## Figures and Tables

**Table 1:** 

Species	ID	Infection	Efficiency of plating (EOP)
*E. aerogenes*	3-SP	+	1.000
*E. aerogenes*	201316724	+	1.002
*E. aerogenes*	2015-301	−	0
*E. aerogenes*	13208	−	0
*E. aerogenes*	A29864	−	0
*E. aerogenes*	A36179	−	0
*E. aerogenes*	AH10	−	0
*E. aerogenes*	AH12	−	0
*E. aerogenes*	AH13	−	0
*E. aerogenes*	AH14	−	0
*E. aerogenes*	AH15	−	0
*E. aerogenes*	AH17	−	0
*E. aerogenes*	AH18	−	0
*E. aerogenes*	AH2	−	0
*E. aerogenes*	AH20	−	0
*E. aerogenes*	AH21	−	0
*E. aerogenes*	AH22	−	0
*E. aerogenes*	AH24	−	0
*E. aerogenes*	AH25	−	0
*E. aerogenes*	AH28	−	0
*E. aerogenes*	AH29	−	0
*E. aerogenes*	AH3	+	0.001
*E. aerogenes*	AH30	−	0
*E. aerogenes*	AH32	−	0
*E. aerogenes*	AH33	−	0
*E. aerogenes*	AH34	−	0
*E. aerogenes*	AH36	−	0
*E. cloacae*	T5282	−	0
*E. cloacae*	TI3	−	0
*E. sakazakii*	45401	−	0
*E. sakazakii*	45402	−	0
*Serratia marcescens*	wk2050	−	0
*S. marcescens*	201315732	−	0
*S. marcescens*	wj-1	−	0
*S. marcescens*	wj-2	−	0
*S. marcescens*	wj-3	−	0
*Escherichia coli*	ATCC 25922	−	0
*Klebsiella pneumoniae*	ATCC BAA-1706	−	0
*Achromobacter xylosoxidans*	A22732	−	0
*Leclercia adcarboxglata*	P10164	−	0
*Raoultella ornithinolytica*	YNKP001	−	0
*Stenotrophomonas maltophilia*	9665	−	0
*Citrobacter freundii*	P10159	−	0
*Vibrio parahaemolyticus*	J5421	−	0
*Pseudomonas aeruginosa*	PA01	−	0
*Acinetobacter baumannii*	N1	−	0
*Shigella sonnei*	#1083	−	0

